# Lecture on Pneumonia—Pathological Anatomy of Pneumonia—Pneumothorax—Pericarditis—Endocarditis—Fatty Liver

**Published:** 1840-01-11

**Authors:** W. W. Gerhard


### CLINICAL LECTURE.

### PHILADELPHIA HOSPITAL.

*Wednesday, January* 1*st*, 1840.

LECTURE ON PNEUMONIA—PATHOLOGICAL ANATOMY OF PNEUMONIA—PNEUMOTHORAX—PERICARDITIS—ENDOCARDITIS—FATTY LIVER.

By W. W. Gerhard, M. D.

*No.* 7—*Winter Course.*

The lecture of to-day, gentlemen, will be principally occupied with the consideration of a disease, which is extremely frequent in the winter and spring months,—I mean pneumonia. We have as yet had no cases of the disease in its pure and regular form; they have all been more or less irregular in their character. For pneumonia has its irregularities of aspect, as well as phthisis; and an attention to these deviations from the regular form, are of extreme importance in a practical point of view.

Pneumonia may assume an irregular character from several causes. 1. Age: thus, in old persons, this disease tends constantly to put on an asthenic form. 2. The previous condition of the system: if the health has been deranged by some antecedent disease, a corresponding modification will be impressed upon the pneumonia. 3. The epidemic constitution of the atmosphere: in consequence of which, this, as well as various other diseases, will, at one time, have a sthenic, at another time, an asthenic character. The mortality of this affection varies exceedingly at different periods of life. In old persons, that is, those past the age of fifty, it is one of the most fatal of all diseases: it is then often characterized by a feeble or asthenic grade of action. In children, also, below the age of six years, pneumonia is extremely fatal; at this age, we have the variety called *lobular*, which usually attacks both lungs. From the sixth or seventh to the forty-fifth year, on the other hand, idiopathic pneumonia, treated at the commencement, is hardly ever fatal; and when death does occur, it may be frequently referred to some impropriety of treatment, or else some modifying circumstances of an unusual kind, which have caused the disease to assume an unfavourable aspect.

Pneumonia is an inflammation of the substance or parenchyma of the lungs,—that is, the vesicular terminations of the bronchial tubes, and the cellular tissue connecting them. The other structures,—the pleural covering, and the bronchial tubes,—are likewise almost invariably implicated. There are three distinct stages in this affection, each dependent upon a corresponding condition of the pulmonary tissue. In the first, or engorged stage, the lung, like any other organ in a state of inflammation, simply contains a larger quantity of blood than natural, mixed with serum; the vesicular structure is still completely preserved. When we make a section of a lung in this state, a bloody serum oozes from the cut surface; it is of a red colour, more firm to the touch than a healthy lung, but more friable. In the second stage, or that of hepatization, the lung becomes impermeable to the air,—its parenchyma is solidified by the blood and lymph which fill it,—the colour varies from one shade of red to another,—the lung is so dense and resisting to pressure, (though still easily broken down into a pulp,) as to bear a striking resemblance to the substance of the liver,—hence the term by which this stage is designated. In the third stage, the blood and lymph disappear, and are replaced by pus, which, like them, is infiltrated through the pulmonary tissue. The divided surface is of a yellowish or grayish colour, and a purulent fluid escapes from it when pressure is made: of course, the vesicular structure is then destroyed. This stage is called gray or yellow hepatization, or softening. The pus is in most cases, as I have stated, diffused throughout the substance of the lung: in some rare instances, however, it collects into an abscess, and its discharge leaves a cavity opening into a bronchial tube. Pneumonia in young or adult subjects, rarely runs through all these stages, but passes into resolution after it has reached the second: in the old, however, it does, in most cases, run through all of them, and often with great rapidity, either to a fatal or a favourable termination.

The case which I now bring before you, is a very good illustration of the course of the disease: but it is not an example of pure pneumonia, because the physical signs, and the history of the case, render it probable that it is complicated with the development of tubercles in the lungs. The patient, 29 years of age, was admitted on the 20th ult. He had been sick for about three weeks. He had usually enjoyed good health, except occasional attacks of fever. Last autumn he had a bilious fever, from the effects of which he had not entirely recovered, when he was seized with the symptoms of pulmonary disease: that is, he was still feeble, and unable to do full work. The first of these symptoms was a chill, followed by cough, which has continued to the present time. There was also pain, which commenced near the right nipple, and passed round to the scapula of the same side. The action of the heart has not been disordered; there was no epistaxis until the 25th, when a small discharge occurred. Nor was there any blood in the expectoration, until the 27th: it was then intimately mixed with the sputa, and not in the distinct form properly denominated hæmoptysis. The patient entered the hospital on the 26th: on the 27th I examined him for the first time, and found the functional and physical signs of pneumonia. There was a circumscribed flush on each cheek, brighter than that of ordinary pneumonia, and nearly resembling that of hectic, about an inch and a half in diameter, and becoming more distinct at each exacerbation of the fever. There was considerable dyspnœa, the nostrils were dilated, and the countenance had that expression of anxiety which always attends difficulty of respiration. The intellect was perfectly clear, and there were no cerebral symptoms. The tongue was moist, and covered with a white coat; appetite bad; bowels were readily opened by a laxative. The cough was shorter than it usually is in pneumonia; expectoration small in quantity, white, and opaque, without any tinge of blood. The respiration on the right side posteriorly, was bronchial, from the middle to the summit of the lung; there was flatness on percussion over the same space, and dulness throughout the rest of the right side. There was also some crepitant rhonchus around the portion of lung which yielded the flatness on percussion, and bronchial respiration. It will be seen from the history of this case, that it differs from ordinary, frank pneumonia in two respects: first, it succeeded an attack of another disease, which had left the patient in a more or less debilitated condition; and secondly, it had more the character of a chronic than of an acute disease. If it had been acute pneumonia, in the three or four weeks which have elapsed from its commencement, the patient would either have died, or been convalescent: its long continuance shows that the grade of action is subacute. This circumstance modifies both our diagnosis and prognosis of the case. It is probable that a development of tubercles in the lungs preceded the pneumonia, and of course our prognosis is on that account the more unfavourable. Since the 27th, the symptoms have gradually declined; the flush on the cheek is less distinct, the skin is moist, the respiration and pulse are becoming natural. The flush is now of a bright red colour; on the 27th, it had a purple tinge, owing to the deficient deterialization of the blood, which necessarily arose from the impermeable condition of the lung.

The pulse becoming slower, is always a good omen in pneumonia, provided that the respiration at the same time approaches the natural standard. But if the respiration become more frequent, at the same time that the pulse sinks, the prognosis is just the reverse. Thus, if to-day the pulse were 140 in the minute, and the respiration 32, and to-morrow they should change, the one to 100, the other to 45, we would infer that the patient's condition was much worse: because the increased frequency of the respiration depends upon the extension of the disease in the lungs; whereas the diminished frequency of the pulse depends upon the action of the heart becoming enfeebled. If on the third day, we should find that the respiration had risen to 56, while the pulse had fallen to 80, we might consider the case hopeless. On the contrary, if while the pulse fell from 140, to 100, and then to 72, the respiration should fall to 32, and finally to 26, the prognosis would be favourable, for both signs would indicate a declension of the pulmonary disease. Hence, in the prognosis of pneumonia, we ought to lay much more stress upon the respiration than the pulse: the latter is in fact almost nugatory as a sign in this disease, and is only of use as indicating the degree of the patient's strength, and the amount of bleeding which he will bear,—which indeed may be inferred nearly as well from other signs, as from the state of the pulse. The respiration indicates precisely in acute cases, the gravity of the affection, for in proportion as the lung becomes solidified, the respiration must of course become more rapid, in order to supply the deficiency of air resulting therefrom. But, in chronic cases, the sign is of less importance, because the patient gradually accustoms himself to a smaller supply of air. In the pneumonia of children, an attention to the respiration is of even greater importance than it is in adults: for in the former it is difficult to ascertain satisfactorily the state of the pulse; whereas the respiration can always be counted with ease. In counting the respiration in patients of any age, we should be careful not to touch the patient, for it is apt to confuse him, and make the respiration in some degree voluntary. In the present case, the respiration is 26, and the pulse 78—a very moderate degree of acceleration. The former, even in adults, often rises to 30 or 40, and is sometimes much more frequent. This, in children, would not be considered as a great degree of frequency: I have sometimes seen the respiration in them more frequent than the pulse: such cases of course must be for the most part, fatal. The physical signs in the case before us, have likewise become more favourable: the bronchial respiration is less intense, and in the region occupied by it, some crepitus is now heard. Indeed, at the last examination that I made of the patient, I found that the respiration was again vesicular, though feeble.

The *treatment* has consisted in the application of six cups to the right side, followed by a blister, and the administration of the following combination:

| | |
|---|---|
| ℞.—Pulv. Ipecac. | gr. j. |
| Calomel, | gr. ¼. |
| Pulv. Opii, | gr. 1-12. M. |

S. To be repeated every three hours. The gums are slightly red, but the ptyalism, if it has occurred at all from the use of this prescription, is extremely slight. In cases of this sort, we must rely upon local depletion, followed sometimes by a blister, and some remedy calculated to allay the excitement of the heart. For this purpose, nothing is better than the old-fashioned combination of opium, calomel and ipecac. Active depletion by venesection cannot be resorted to, and sometimes even cupping is contra-indicated; in such cases, blisters will answer exceedingly well.

The next case is an illustration of the manner in which pneumonia is modified by age, and the epidemic tendency of the season: it is one of subacute, or asthenic pneumonia, passing into gangrene, the supervention of the latter being indicated by a change in the expectoration, and the sinking of the patient. The patient, aged 54, was admitted December 18th. He had previously enjoyed good health, with the exception of an attack of intermittent last autumn, which lasted three months, but was finally cured. Two months since a cough commenced, but there was no pain till a fortnight ago, when it was felt in the right axilla. The disease was, therefore, in all probability, bronchitis in the commencement, and the pneumonia did not take place till about the period of of his admission into the hospital. The expectoration was then whitish and small in quantity; it has since been yellow and purulent, sometimes mixed with blood. The patient lay on the right side; he was unable to lie on the left, on account of the pain experienced in that position. The action of the heart was normal. On the 22d, the patient exhibited the signs of ordinary pneumonia. There was dyspnœa; hard and dry cough, with yellow and puriform expectoration; tongue reddish; no pain in epigastrium. Percussion dull on the right side in the lower third of the lung; with mucous and crepitant rhonchi; the respiration had lost its vesicular character. 24th. Respiration more rapid than before; pulse less so; expectoration viscid and red. As the patient appeared to be sinking, he was put on the use of wine whey and sanguinaria. In the afternoon of the same day, there were signs of gangrene supervening; the sputa had a gangrenous odour, and amounted to eight ounces in the course of twenty-four hours; they were of a dark colour, tinged with blood, and resembled the jelly of Iceland moss; pulse 84, respiration 25; patient very weak; carbonate of ammonia and Hoffman's anodyne were ordered. A cavity was gradually formed by the progress of the gangrene, as was proved by a mucous rhonchus so loud and loose as almost to constitute gurgling. This case illustrated the rapidity with which the vitality of the pulmonary tissue may be destroyed; the change in the character of the sputa, and the prostration of strength occurred suddenly. The symptoms had previously been gradual in their occurrence. The sputa have at no time presented the characters which they do in ordinary pneumonia; they were mucous previously to the occurrence of the gangrene, with only a slight degree of viscidity and intermixture of blood. They now consist of a floculent, shreddy matter floating in a fluid; a few days since they were very dark, but are now less so; the gangrenous fetor has also, in a great measure, disappeared, and there is an intermixture of pus. This last circumstance is a proof that the progress of the gangrene has been arrested, and a complete cure of the lesion will be effected by means of a false membrane, (in the manner which I have already explained to you,) unless the sloughing should again commence. This occurrence, of course, would be indicated by an increase of the mucous rhonchus passing into the cavernous, with fetor and dark colour of the sputa; With regard to the gangrene, our prognosis is, therefore, in the present case, favourable. Generally, gangrene of the lungs is less fatal in private practice than in hospitals, because, in the former case, our means of supporting the patient by a proper diet are much less limited than in the latter.

In the asthenic variety of pneumonia, the three stages are usually completed with great rapidity; the danger, however, is not from the implication of a large portion of the pulmonary tissue, so much as from the loss of strength and inability to expectorate, which constantly takes place. Hence all depletion, except by a few cups, is contra-indicated; we have, on the contrary, to support the patient and to promote expectoration. For the latter purpose, we have employed very effectually, an infusion of sanguinaria and senega. The formula is as follows:

℞.—Rad. Sanguinariæ Canad., ʒij.

Rad. Polyg. Senegæ, ʒiv.

Aquæ ferv. Oj.—Ft. In. S. To be be taken in divided doses in 24 or 48 hours, as the stomach will bear it.

Or we may employ the combination of calomel, opium and ipecac. already spoken of, particularly if the inflammation should have some degree of activity. Blisters are likewise very useful in certain cases. The difference of opinion which exists as to their utility, has doubtless arisen from a want of due discrimination as to the character of the disease, and the proper period for their application. In frank pneumonia, where the grade of action is high, and the inflammation does not usually pass beyond the second stage, they will indeed prove injurious, unless preceded by sufficient depletion; but even in these cases, if they should pass into the third stage, blisters are necessary items in the treatment. In the asthenic variety of the disease, where the vitality of the lungs is impaired, and the grade of action is weak, blisters are exceedingly useful, and they are generally tolerated very easily.

The next patient, S. C., you have already seen; he has been labouring under a chronic disease of the lungs, complicated with disease of the heart, which is now declining. The first symptom in this case was cough, which continued for some time, when it was accompanied by dyspnœa, and pain in the præcordial region. On the 24th of December, when he was admitted into the hospital, the sounds of the heart were clear, but there was a creaking sound heard during the diastole; there was also dulness on percussion. I hence inferred that there was pericarditis, with some effusion; while the cough and other signs proved the coexistence of phthisis. The treatment has consisted in the application of a blister to the præcordial region, together with the use of a combination of digitalis, calomel, and Dover's powder. Under this treatment, all the acute symptoms have rapidly declined.

I have several pathological specimens to show you to-day, some of which possess considerable interest. The first is the pericardium and heart of a negro, who died within twenty-four hours after his entrance into the hospital. Previously to his entrance, he had been utterly neglected. No very accurate examination of the physical signs could be made, but I recollect that there was an enlargement in the cardiac region, with flatness on percussion: the sounds of the heart were distant and indistinct, and the impulse was feeble: there was likewise some dulness on percussion on the right side of the chest posteriorly. The man had been ill two weeks with pain in the region of the heart. From these signs I inferred that there was pericarditis with effusion, and that the lung and pleura of the right side had become involved in the disease. The causes of pericarditis, when acute, are either the general causes of serous inflammation, as cold, mechanical violence, &c., or rheumatism: the result is, an inflammatory effusion. When chronic, it terminates in an effusion of that sort which constitutes a dropsy, (hydro-pericardium,) in the same way that hydro-thorax sometimes results from chronic pleuritis. The chronic inflammation may follow the acute, or the effusion may result from valvular disease of the heart, or from disease of the liver or kidneys.

When the heart was removed from the body, the flatness on percussion was explained by the discovery of an extraordinary effusion into the pericardium, which was immensely distended, so as to measure 7⅝ inches in length, and 7 in breadth. It contained about a quart of well formed *pus.* The heart is of a much smaller size than natural, owing obviously to the compressing force exercised by the large quantity of fluid by which it was surrounded, in the same manner that the lung is compressed by pleuritic effusions. The pus was mixed with lymph, some of which floated in the fluid, while the rest covered the two surfaces of the pericardium. In one point, adhesion has already taken place, by means of bands which are completely organized: around these is a quantity of lymph more or less hardened, and in different degrees of organization. The question as to the thickening of serous membranes may be considered as settled by this case. If we consider simply the varnished surface as the serous membrane, of course it does not admit of thickening, but if we include in it, (as we ought to, certainly,) all that layer of condensed cellular tissue, which really constitutes the membrane, and presents a smooth surface on the interior, there can be no doubt on the subject. Here you see the membrane greatly thickened, and of an almost cartilaginous density. No creaking sound in the diastole of the heart was heard when the patient was examined, because the expansion of the organ was in a great measure prevented by the effusion: it is only heard when there is a moderate quantity of lymph in the pericardium, and no effusion sufficient to produce compression of the heart. Pericarditis, like pneumonia, is very rarely fatal: when patients affected with it die, it is generally owing to the supervention of endocarditis, a much more fatal disease. This point is perfectly well ascertained. In this case, however, the pericarditis resulted in the death of the patient: I have not examined the interior of the heart, to see whether it was complicated with endocarditis, but most probably it was not; at least, I can feel the valves, which are nearly natural. The pericarditis was too intense; it is only when each exists in a moderate degree, that we commonly find these two diseases together.

Here is another heart, which affords an example of endocarditis; and the specimen is curious, insomuch as it shows that the disease existed in the right side of the heart, instead of the left, the usual seat. The result of the inflammation is a thickening of the tricuspid valve, and a cartilaginous deposit on its margin.

You will recollect that a few weeks since I showed you a coloured man, in whom that lesion which is termed pneumothorax, had occurred. He has since died from the effects of the accident, and I now exhibit to you the lung which was affected. The patient had the symptoms of phthisis before the occurrence of the pneumothorax,—and the perforation of the pleura was undoubtedly caused by a tuberculous cavity seated under this membrane. It was followed by inflammation of the pleura, which resulted in a large effusion of pus. The effusion was so great as to threaten suffocation: the operation of paracentesis was, therefore, performed, which relieved the patient for the time, but hectic fever supervened, and carried the patient off. This result we are always to be prepared for in performing the operation for empyema: we can only rescue the patient from impending suffocation, but we must not be confident of a final cure.

On examining the pleura, no signs of perforation are to be discovered: the reason of this is, that a long time had elapsed since the occurrence of the lesion, and given abundant opportunity for the closure of the opening by coagulable lymph, in the manner which I explained to you in my lecture on this subject. This process was facilitated by the effusion, which compressed the lung, and reduced the size of the opening in the pleura. The lung is much condensed, from the pressure of the liquid; the pleura is injected, and covered with false membrane; tubercles are found in the upper lobe,—and it was doubtless in this portion of the lung that the perforation took place, for no where else can we suppose a cavity to have existed.

Pneumothorax is not necessarily fatal, though it most frequently is so. It may prove fatal, either by the excessive dyspnœa which it produces, or by the effects of the consequent pleurisy.

Here is another lung, which offers an example of the asthenic variety of pneumonia. The subject was an old man, of intemperate habits: when he entered the hospital, he presented the physical signs of pneumonia. The disease proved fatal in a very few days; the brain was likewise involved, and delirium was a prominent symptom of the case. The lung is firm to pressure, and impervious to air; it is of a grayish yellow colour, showing that the disease was passing into the third stage,—and it is at this point of its progress that death usually occurs. When pressure is made, pus, mixed with mucus, oozes from the cut surface,—but no air can be detected in the fluid. Little granular elevations are perceived at various points: these are the vesicles, distended by the morbid matter; they contain no air. The pus is not collected into an abscess, but is infiltrated through the substance of the lung; the tissue is friable, and sinks at once in water. Another portion of the lung has the characters of the second stage of pneumonia: it is dense and resisting, of a red colour, and equally impervious to air with the portion already described. At the summit again, we find a part in the first stage of inflammation; it is infiltrated with blood and serum, but still contains air, which escapes in bubbles where pressure is made. The preceding conditions of the lung correspond with the physical signs which existed during life: over the hepatized portion the respiration was bronchial; at the summit it was still vesicular, but mixed with the crepitant rhonchus. The bronchial tubes are also inflamed. This inflammation sometimes precedes, and sometimes follows that of the substance of the lung. When bronchitis comes first, it will be indicated by a loose cough, and mucous expectoration: the supervention of pneumonia will be announced by chills, pain in the chest, bronchial respiration, &c. When pneumonia is the original disease, it will manifest itself by the ordinary physical signs, together with dyspnœa and prostration of strength. The mucous membranes of the bronchial tubes in this lung are red, thickened, and covered with mucus. The other lung preserves the healthy vesicular structure, in accordance with the general rule that pneumonia attacks only one lung at a time. An exception to the normal condition of the lung exists, however, at the summit: we there perceive the evidences of a cicatrized tubercular cavity. The lung is puckered by the cicatrix; and gray granulations are seen around it, which are as hard as cartilage, and probably of an indolent character: as the cicatrix has remained unbroken till this advanced age, the cure may be considered complete. In the neighbourhood of the tubercles, the pulmonary tissue is emphysematous: the enlargement of the vesicles can be detected by the eye. This condition was manifested during life by preternatural resonance on percussion, and feebleness of respiration. There is a slight congestion of the lower lobe of the lung, but it is hardly sufficient to constitute a state of disease: the pleura is entirely healthy.

The liver of the same subject is in that state which is termed *fatty degeneration*, and which depends on the deposition of adipose matter in the substance of the organ, especially in the cellular tissue uniting the acini. This change is most commonly met with in intemperate subjects, and those labouring under phthisis, particularly females. The liver is enlarged, of a much lighter colour than natural, and the oily matter exudes from the cut surface, when the back of the scalpel is passed over it. The causes of this alternation, and its precise importance as a pathological state, are by no means understood. The morbid conditions of the liver still offer a wide field for investigation. An idea of the obscurity still subsisting in relation to this subject, may be formed from the statement, that in France, scarcely any attention is paid to the disorders of the liver, while in some portions of the United States, this organ is considered by many as concerned in almost every disease. The reason of this difference of opinion is, that in the temperate parts of Europe, its lesions are rarely of that violent character which naturally excites the attention of medical men. In our Southern States, on the other hand, they are among the most frequent and serious of the diseases incident to that region. Of course, on this, as on every other subject, there is a proper medium; but before it can be accurately defined, future investigations must explain to us, more perfectly than we now understand them, the causes, the nature, and the pathological value of the various alterations to which the liver is subject.

